# Three *Babesia* species in *Ixodes ricinus* ticks from migratory birds in Sweden

**DOI:** 10.1186/s13071-021-04684-8

**Published:** 2021-04-01

**Authors:** Peter Wilhelmsson, Olga Pawełczyk, Thomas G. T. Jaenson, Jonas Waldenström, Björn Olsen, Pia Forsberg, Per-Eric Lindgren

**Affiliations:** 1 Division of Inflammation and Infection, Department of Biomedical and Clinical Sciences, Linköping University, Linköping, Sweden; 2 Department of Clinical Microbiology, Region Jönköping County, Jönköping, Sweden; 3 Department of Parasitology, Faculty of Pharmaceutical Sciences in Sosnowiec, Medical University of Silesia, Katowice, Poland; 4 Department of Organismal Biology, Evolutionary Biology Centre, Uppsala University, Uppsala, Sweden; 5 Center for Ecology and Evolution in Microbial Model Systems, Linnaeus University, Kalmar, Sweden; 6 Zoonosis Science Center, Department of Medical Sciences, Uppsala University, Uppsala, Sweden; 7 Division of Infectious Diseases, Department of Biomedical and Clinical Sciences, Linköping University, Linköping, Sweden

**Keywords:** *Babesia capreoli*, *Babesia microti*, *Babesia venatorum*, *Ixodes ricinus*, Migratory birds, Tick-borne diseases, Sweden

## Abstract

**Background:**

Migratory birds can cross geographical and environmental barriers and are thereby able to facilitate transmission of tick-borne pathogens both as carriers of infected ticks and as reservoirs of pathogenic microorganisms. *Ixodes ricinus* is one of the most abundant tick species in the Northern Hemisphere and a main vector of several *Babesia* species, some which pose a potential threat to human and animal health. At present only two cases of overt babesiosis in humans have so far been reported in Sweden. To better understand the potential role of birds as disseminators of zoonotic *Babesia* protozoan parasites, we investigated the presence of *Babesia* species in ticks removed from migratory birds.

**Methods:**

Ticks were collected from birds captured at Ottenby Bird Observatory, south-eastern Sweden, from March to November 2009. Ticks were molecularly identified to species, and morphologically to developmental stage, and the presence of *Babesia* protozoan parasites was determined by real-time PCR.

**Results:**

In total, 4601 migratory birds of 65 species were examined for tick infestation. Ticks removed from these birds have previously been investigated for the presence of *Borrelia* bacteria and the tick-borne encephalitis virus. In the present study, a total of 1102 ticks were available for molecular analysis of *Babesia* protozoan parasites. We found that 2.4% of the ticks examined, all *I. ricinus*, were positive for mammal-associated *Babesia* species. Out of all *Babesia*-positive samples, *Babesia venatorum* was the most prevalent (58%) species, followed by *Babesia microti* (38%) and *Babesia capreoli* (4.0%). *B. venatorum* and *B. capreoli* were detected in *I. ricinus* larvae, whereas *B. microti* was only present in *I. ricinus* nymphs. This supports the view that the two first-mentioned species are vertically (transovarially) transmitted in the tick population, in contrast to *B. microti*. The largest number of *Babesia*-infected ticks was removed from the common redstart (*Phoenicurus phoenicurus*) and European robin (*Erithacus rubecula*).

**Conclusions:**

This study reveals that *Babesia* protozoan parasites are present in ticks infesting migratory birds in south-eastern Sweden, which could potentially lead to the dissemination of these tick-borne microorganisms into new areas, thus posing a threat to humans and other mammals.

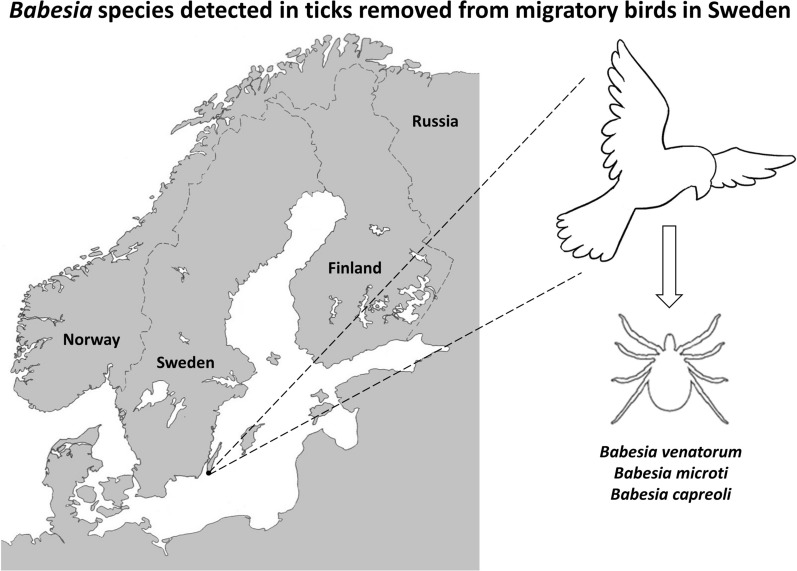

**Supplementary Information:**

The online version contains supplementary material available at 10.1186/s13071-021-04684-8.

## Background

The role of migratory birds as hosts of disease vectors such as ticks and of potentially human pathogenic microorganisms has been increasingly recognized. Birds can cross geographical and environmental barriers and take part in the dispersal of bacteria, viruses, and protozoa [1]. Seasonal migration is especially pronounced in northern Europe, where a large part of the avifauna migrate, either to milder regions in western Europe or the Mediterranean region, or long distances to sub-Saharan Africa or even Asia. This means that birds returning in spring could carry ticks from regions that better sustain year-round transmission of tick-borne pathogens, and possibly contribute to reseeding foci of infections in temperate and boreal areas. Indeed, several studies in the Scandinavian countries have shown the presence of ticks and tick-borne infections in returning migratory birds, especially well-studied pathogens such as *Borrelia burgdorferi* sensu lato (s.l.) [2], *Rickettsia* spp. [3], and tick-borne encephalitis (TBE) virus [4]. However, the potential for less-studied, rarer, or unknown pathogens in bird-borne ticks to be detected in birds has come into focus recently, for instance with the bacterium *Neoehrlichia mikurensis*, which once identified has been shown to be fairly abundant in ticks [5]. Another pathogen of concern is *Babesia* spp., a protozoan parasite causing babesiosis—an emerging tick-borne human disease in the Holarctic region.

More than 100 species of *Babesia* have been described. The majority have been recorded in mammals, and 16 species have so far been described from avian hosts [6, 7]. In Europe, the roe deer (*Capreolus capreolus*) is considered the main vertebrate host of both *Babesia capreoli* and *Babesia venatorum* [8, 9], while *Babesia divergens* is prevalent in cattle in southern Sweden [10, 11]. Serological studies indicate that another species of *Babesia—Babesia motasi*—may be common in sheep herds in south-eastern Sweden [12]. Several of the mammal-associated species, such as *B. divergens*, *Babesia duncani*, *B. venatorum*, and *Babesia microti*, are zoonotic pathogens and of increasing medical importance, causing from asymptomatic infections to mild or serious, sometimes even fatal, human disease, particularly in immunocompromised persons [13–18]. In Europe, most of the severe cases of human babesiosis have involved infections of *B. divergens* [14, 15, 18–20]. However, the relatively recent discovery of the closely related *B. venatorum* [21, 22] may suggest that some of the earlier cases diagnosed as due to *B. divergens* may in fact have been caused by *B. venatorum.* During the last decades, the recorded incidence of human babesiosis due to *B. microti*, which is present in a reservoir of small mammals in the Holarctic region, has increased considerably in the north-eastern United States [23]. A few cases of human disease caused by *B. microti* or *B. microti-*like species have recently also been diagnosed in Europe and Asia [19, 24].

In nature, most mammalian *Babesia* spp. are transmitted by ixodid ticks [7, 25]. However, there is circumstantial evidence suggesting that some of the avian *Babesia* spp. may be vectored by soft ticks [7]. In North America, blood transfusion is recognized as an increasing and serious mode of accidental transmission of *B. microti* [26]. Human *B. microti* infection by blood transfusion has also been documented in Europe [24].

In Sweden, *Ixodes ricinus* is the most abundant tick species infesting humans [27], and also the most abundant tick species detected on passerine birds during migration in southern Sweden [2]. Three species of potentially zoonotic *Babesia* species*—B. divergens*, *B. microti*, and *B. venatorum*—have been recorded at prevalence rates of 0.2%, 3.2%, and 1.0%, respectively, in questing *I. ricinu*s ticks in southern Sweden [28]. In a study that examined 2038 *I. ricinus* ticks which had been removed from humans in Sweden and on the Åland Islands, Finland, *B. capreoli*, *B. microti*, and *B. venatorum* were recorded in 0.25%, 1.60%, and 1.30% of the ticks, respectively [29].

The aim of this study is to better understand the potential role of migratory birds as disseminators of *Babesia* protozoan parasites and to determine the prevalence of *Babesia* spp. in ticks infesting birds during their spring and autumn migrations in south-eastern Sweden. Part of this study, related to data on the prevalence of *Borrelia* spp. and the TBE virus in ticks removed from migratory birds during 2009, has been reported elsewhere [30].

## Methods

### Sampling, analyses, and processing of ticks

Full details of the sampling site, procedure for bird captures and bird classification, determination of developmental stage and species of collected ticks, and total nucleic acid extraction from ticks and cDNA synthesis are available in the previous report [30].

In short, ticks were collected from birds captured during the periods 15 March–15 June and 15 July–15 November 2009 at the Ottenby Bird Observatory, which is located on the southern point of the island of Öland in south-eastern Sweden (56° 12′ N, 16° 24′ E). Trapped birds were identified to species level and classified into residents, short-distance migrants, partial migrants, and long-distance migrants. Any collected tick was photographed and morphologically identified to stage of development (larva, nymph, or adult), and sex of adults. Each tick was individually homogenized using a TissueLyser II (Qiagen) followed by extraction, purification, and isolation of total nucleic acids using MagAttract^®^ Viral RNA M48 kit in a BioRobot M48 workstation (Qiagen). The total nucleic acids were reverse-transcribed to cDNA using the illustra™ Ready-to-Go RT-PCR Beads kit (GE Healthcare, Amersham Place, UK), which served as template in all the polymerase chain reaction (PCR) assays. To identify the genus and species of the ticks, each specimen was analysed by a PCR method targeting the tick mitochondrial *16S* rRNA gene followed by DNA sequencing, as previously described [30].

Sampling of birds was approved by the Swedish Board of Agriculture, delegated through the Animal Research Ethics Committee in Linköping (decision 43–09).

### Detection and determination of *Babesia* species

Detection of *Babesia* spp. was done using a SYBR green real-time PCR assay, as previously described [10]. Primers BJ1 (5′–GTC TTG TAA TTG GAA TGA TGG–3′) and BN2 (5′–TAG TTT ATG GTT AGG ACT ACG–3′) were designed to target the *Babesia 18S* rRNA gene to amplify a 411–452-bp long amplicon depending on the species of *Babesia* [31].

A 20-μl reaction consisted of 10 µl SYBR™ Green PCR Master Mix (Thermo Fisher Scientific, Stockholm, Sweden), 0.4 µl of each primer (10 µM; Invitrogen), 6.2 µl RNase-free water, and 3 µl cDNA template. The PCR template of 3 µl consisted of a pool of cDNA from three tick specimens per reaction. A positive PCR control, consisting of 3 µl *B. microti* DNA (10 ng/µl) extracted from an *I. ricinus* tick collected in Slovakia, was included in each run. The *B. microti* DNA was kindly provided by Dr Bronislava Víchová (Institute of Parasitology, Slovak Academy of Sciences, Slovakia) through Dr Martin Andersson (Centre for Ecology and Evolution in Microbial Model Systems, Linnaeus University, Kalmar, Sweden). As a negative control in the PCR assay, RNase-free water was used as template. When *Babesia*-positive pools were detected, the samples were re-analysed individually.

The PCR reactions were performed on a C1000™ Thermal Cycler, CFX96™ Real-Time PCR Detection System (Bio-Rad Laboratories, Inc., Hercules, CA, USA) using an activation step at 94 °C for 10 min, and 35 cycles of 94 °C for 1 min, 55 °C for 1 min, and 72 °C for 2 min, and finally one cycle of 72 °C for 5 min. Immediately after completion of PCR, melting curve analyses were performed by heating to 95 °C for 15 s, followed by cooling to 60 °C for 1 min, and subsequent heating to 95 °C at 0.8 °C min^−1^ with continuous fluorescence recording.

To determine the species of *Babesia* in the PCR-positive samples, nucleotide sequencing of the PCR-products was performed by Macrogen Inc. (Amsterdam, The Netherlands). All sequences obtained were confirmed by sequencing both strands. The obtained chromatograms were initially edited and analysed using BioEdit Software v7.0 (Tom Hall, Ibis Therapeutics, Carlsbad, CA, USA), and the sequences were examined using the Basic Local Alignment Search Tool (BLAST). Sequences obtained have been deposited in GenBank with accession numbers ranging from MW554592 to MW554617.

Species determination of *B. microti* and *B. venatorum* is possible by sequencing the amplicon from the real-time PCR assay. *B. capreoli* is highly similar to *B. divergens*, and the two species differ only at three nucleotide positions at the *18S* rRNA gene, specifically on positions 631, 663, and 1637 (99.83% nucleotide similarity) [32]. The two first positions are included in the DNA fragment amplified by the primers used in this study. Additional file 1 shows all the aligned *Babesia* nucleotide sequences.

### Statistical analyses

Data were presented as percentages for categorical variables. The categorical variables were analysed using the chi-square test, but when the expected frequency was < 5 in at least one of the cells of the contingency table, Fisher’s exact test was used. Statistical analyses were performed using GraphPad Prism version 8.0.0 for Windows (GraphPad Software, San Diego, CA, USA). *P* values ≤ 0.05 were considered statistically significant.

## Results

### Ticks collected from birds and ticks available for PCR analyses

A total of 4601 bird individuals (4788 bird captures) of 65 species were examined for ticks at least once during the study period at the Ottenby Bird Observatory. A total of 749 bird individuals (759 bird captures) of 35 species were infested with a total of 1339 ticks (Table 1). These results were previously reported in the study of Wilhelmsson et al. [30].

**Table 1:** Ticks collected from bird species at Ottenby Bird Observatory, south-eastern Sweden, 2009 [30]

Bird species	Migratory category^a^	No. of bird captures	No. of infested birds (%)	No. of ticks	Mean no. ticks per infested bird ± SE^d^	Median no. ticks per infested bird (IQR)^d^	Tick species and developmental stage^c^					
							*Ixodes* spp.	*I. r*	*I. f*	*H. p*	*Hy. m*	ND
*Accipiter nisus*	PM	5	1 (20)	1	–	–		1 N				
*Acrocephalus palustris*	LM	21	3 (14)	4	1.3 ± 0.33	1 (1.00–2.00)		4 N				
*Acrocephalus scirpaceus*	LM	11	1 (9.0)	1	–	–		1 N				
*Anthus trivialis*	LM	39	26 (67)	93	3.6 ± 0.73	2 (1.00–4.25)	4L	52L, 33 N				4
*Carduelis cannabina*	SM	15	1 (6.7)	1	–	–				1L		
*Carduelis flammea*	SM	36	2 (5.6)	7	3.5 ± 1.50	3.5		7 N				
*Certhia familiaris*	PM	26	2 (7.7)	4	2.0 ± 0.00	2		2L				2
*Cyanistes caeruleus*	PM	58	4 (6.9)	7	1.6 ± 0.75	1 (1.00–3.25)		1 N		4L		2
*Emberiza schoeniclus*	SM	28	2 (7.1)	3	1.5 ± 0.50	1.5		2 N				1
*Erithacus rubecula*	SM	1551	368 (24)	619	1.7 ± 0.09	1 (1.00–2.00)	38L, 13 N	278L, 194 N, 3U	5L, 11 N, 1A		1L	75
*Fringilla coelebs*	SM	37	6 (16)	8	1.3 ± 0.21	1 (1.00–2.00)		4L, 2 N			1A	1
*Fringilla montifringilla*	SM	8	1 (13)	1	–	–		1L				
*Hippolais icterina*	LM	50	2 (4.0)	2	1.0 ± 0.00	2		1L, 1 N				
*Lanius collurio*	LM	43	3 (7.0)	3	1.0 ± 0.00	1 (1.00–1.00)		3 N				
*Luscinia luscinia*	LM	18	10 (56)	29	2.9 ± 0.78	2 (1.75–3.25)		20L, 9 N				
*Luscinia svecica*	LM	28	2 (7.1)	2	1.0 ± 0.00	1	1 N	1 N				
*Motacilla alba*	LM	23	1 (4.3)	3	–	–		2L		1L		
*Oenanthe oenanthe*	LM	6	1 (17)	1	–	–				1L		
*Parus major*	PM	44	3 (6.8)	8	2.7 ± 1.67	1 (1.00–6.00)		2 N				6
*Passer domesticus*	R	16	1 (6.3)	1	–	–		1 N				
*Phoenicurus ochruros*	LM	10	2 (20)	2	1.0 ± 0.00	1		2 N				
*Phoenicurus phoenicurus*	LM	134	40 (30)	64	1.6 ± 0.22	1 (1.00–2.00)		29L, 32 N				3
*Phylloscopus collybita*	LM	79	3 (3.8)	3	1.0 ± 0.00	1 (1.00–1.00)		2 N				1
*Phylloscopus trochilus*	LM	810	38 (4.7)	57	1.5 ± 0.14	1 (1.00–2.00)	1L, 1 N	19L, 30 N	1L		2 N	3
*Prunella modularis*	SM	44	16 (36)	24	1.5 ± 0.14	1 (1.00–1.00)	2 N	3L, 19 N				
*Regulus regulus*	PM	319	9 (2.8)	9	1.0 ± 0.00	1 (1.00–1.00)		2L, 7 N				
*Sturnus vulgaris*	SM	14	3 (21)	3	1.0 ± 0.00	1 (1.00–1.00)		2 N		1L		
*Sylvia atricapilla*	LM	58	4 (6.9)	4	1.0 ± 0.00	1 (1.00–1.00)		2L, 1 N				1
*Sylvia communis*	LM	123	27 (22)	49	1.8 ± 0.37	1 (1.00–2.00)		23L, 17 N		4L		5
*Sylvia curruca*	LM	270	10 (3.7)	11	1.1 ± 0.10	1 (1.00–1.00)		7L, 2 N				2
*Troglodytes troglodytes*	PM	254	72 (28)	120	1.7 ± 0.20	1 (1.00–2.00)	13L, 2 N	59L, 31 N, 1U	1L			13
*Turdus iliacus*	SM	29	9 (31)	23	2.6 ± 0.69	2 (1.00–4.50)		1L, 18 N	1 N			3
*Turdus merula*	PM	147	66 (45)	149	2.3 ± 0.25	1 (1.00–3.00)	6 N	8L, 108 N, 1U	1L, 2 N			23
*Turdus philomelos*	SM	60	9 (15)	22	2.4 ± 0.58	2 (1.00–3.50)	2 N	2L, 17 N	1 N			
Unknown^b^		1	1 (100)	1	–	–		1 N				
*Acrocephalus arundinaceus*	LM	1	0	0								
*Acrocephalus schoenobaenus*	LM	23	0	0								
*Anthus pratensis*	SM	5	0	0								
*Carduelis carduelis*	SM	15	0	0								
*Carduelis chloris*	SM	16	0	0								
*Carduelis spinus*	SM	9	0	0								
*Carpodacus erythrinus*	LM	2	0	0								
*Coccothraustes coccothraustes*	PM	1	0	0								
*Corvus monedula*	R	1	0	0								
*Delichon urbica*	LM	43	0	0								
*Dendrocopos major*	PM	1	0	0								
*Emberiza citrinella*	SM	23	0	0								
*Ficedula albicollis*	LM	2	0	0								
*Ficedula hypoleuca*	LM	31	0	0								
*Ficedula parva*	LM	8	0	0								
*Hirundo rustica*	LM	25	0	0								
*Jynx torquilla*	LM	1	0	0								
*Locustella naevia*	LM	1	0	0								
*Motacilla flava*	LM	2	0	0								
*Muscicapa striata*	LM	55	0	0								
*Passer montanus*	R	37	0	0								
*Phylloscopus borealis*	LM	1	0	0								
*Phylloscopus sibilatrix*	LM	19	0	0								
*Picus viridis*	R	1	0	0								
*Pyrrhula pyrrhula*	PM	2	0	0								
*Saxicola rubetra*	LM	1	0	0								
*Serinus serinus*	SM	2	0	0								
*Sylvia borin*	LM	42	0	0								
*Sylvia nisoria*	LM	1	0	0								
*Turdus pilaris*	SM	1	0	0								
*Turdus viscivorus*	SM	1	0	0								
Total number of specimens		4788	749	1339	–		56L, 27 N	515L, 551 N, 5U	8L, 15 N, 1A	12L	1L, 2 N, 1A	145

^a^*LM* long-distance migrants (in many cases wintering in Africa), *SM* short-distance migrants where the majority of individuals winter in Europe, *PM* partial migrants where individuals either migrate short distances within Europe or remain resident, *R* residents

^b^Probably *Lanius collurio*

^c^*L* larva *N* nymph, *A* adult female, *U* unknown developmental stage due to missing photos, *I. r.* *Ixodes ricinus*, *I. f.*, *I. frontalis*, *H. p., Haemaphysalis punctate, Hy. m., Hyalomma marginatum*, *ND* not determined to genus, species, or developmental stage due to missing photos of ticks and/or loss of sample

^d^*SE* standard error,* IQR* interquartile range

In this study, 1102 ticks (i.e., cDNA samples) were available for analysis. Of these, 543 were larvae, 552 nymphs and two ticks were adult females. Five ticks could not be determined to developmental stage due to lack of photos. The ticks available for analysis were molecularly identified to: *I. ricinus* (*n* = 1,051; 514 larvae, 532 nymphs, 5 specimens of unknown developmental stage), *I. frontalis* (*n* = 24; 8 larvae, 15 nymphs, 1 adult female), *Haemaphysalis punctata* (*n* = 12; 12 larvae), and *Hyalomma marginatum* (*n* = 4; 1 larva, 2 nymphs, 1 adult female). The remaining 11 ticks could not be molecularly identified to species due to unreadable sequences despite several sequencing attempts. Instead, these 11 ticks were only determined to genus level, *Ixodes* (8 larvae, 3 nymphs), based on the photos.

### Prevalence of *Babesia* species in ticks removed from birds

Of the ticks analysed, 2.4% (26/1,102) were positive for *Babesia* spp. All *Babesia-*positive ticks belonged to *I. ricinus* (26/1,051). Of these, 2.0% of the *I. ricinus* larvae were *Babesia*-positive in the PCR-assay (10/514) and 3.0% of the *I. ricinus* nymphs were *Babesia*-positive (16/532). All five *I. ricinus* ticks that could not be determined to developmental stage were *Babesia*-negative. No significant difference in *Babesia* prevalence between larvae and nymphs was detected.

Three species of *Babesia* were identified: *B. venatorum* (*n* = 15), *B. microti* (*n* = 10), and *B. capreoli* (*n* = 1). *B. venatorum* was detected in 9 larvae and 6 nymphs, *B. microti* in 10 nymphs, and *B. capreoli* in 1 larva (Table 2).

**Table 2:** Prevalence of *Babesia* species in *Ixodes ricinus* ticks removed from birds captured at the Ottenby Bird Observatory, Sweden 2009

Tick developmental stage	No. of ticks examined^a^	No. (%) of *Babesia*-positive ticks	No. of ticks containing *Babesia* species determined by nucleotide sequencing		
			*Babesia venatorum*	*Babesia microti*	*Babesia capreoli*
Larva	514	10 (2.0)	9		1
Nymph	532	16 (3.0)	6	10	
Unknown^b^	5	0 (0.0)			
Total	1051	26 (2.5)	15	10	1

^a^All ticks were examined for *Babesia* spp. by a real-time PCR assay

^b^Ticks could not be identified to developmental stage due to missing photos

Of all samples that were determined to *Babesia* species level (*n* = 26; Table 2), 17 were detected in ticks captured in spring (15 March–15 June), and 9 were detected in ticks in late summer–autumn (15 July–15 November). No significant difference was detected between the proportions of *Babesia*-positive ticks collected in spring (17/495) and *Babesia*-positive ticks collected in late summer–autumn (9/556).

### Bird species with ticks positive for *Babesia* species

*Babesia*-positive *I. ricinus* ticks were removed from eight bird species in spring or in late summer–autumn (Table 3): the common redstart (*Phoenicurus phoenicurus*, *n* = 7), European robin (*Erithacus rubecula*, *n* = 7), common blackbird (*Turdus merula*, *n* = 5), Eurasian wren (*Troglodytes troglodytes*, *n* = 2), tree pipit (*Anthus trivialis*, *n* = 2), common starling (*Sturnus vulgaris*, *n* = 1), common whitethroat (*Sylvia communis*, *n* = 1), and lesser whitethroat (*Sylvia curruca*, *n* = 1).

**Table 3:** Species of *Babesia* identified in *Babesia*-positive, immature *Ixodes ricinus* ticks removed from bird species

Bird species	*Babesia*-positive ticks (spring/autumn)^a^	*Babesia venatorum *(larvae/nymphs)	*Babesia microti *(larvae/nymphs)	*Babesia capreoli *(larvae/nymphs)
*Phoenicurus phoenicurus*	7 (5/2)	2 (2/–)	4 (–/4)	1 (1/–)
*Erithacus rubecula*	7 (5/2)	6 (4/2)	1 (–/1)	
*Turdus merula*	5 (2/3)	3 (1/2)	2 (–/2)	
* Troglodytes troglodytes*	2 (1/1)	2 (2/–)		
*Anthus trivialis*	2 (2/–)		2 (–/2)	
*Sturnus vulgaris*	1 (1/–)	1 (–/1)		
*Sylvia communis*	1 (1/–)	1 (–/1)		
*Sylvia curruca*	1 (–/1)		1 (–/1)	
Total	26 (17/9)	15 (9/6)	10 (–/10)	1 (1/–)

^a^*Babesia*-positive *I. ricinus* ticks were removed from birds during 15 March–15 June and 15 July–15 November 2009 at Ottenby Bird Observatory, south-eastern Sweden

## Discussion

So far, only a few studies have investigated tick-borne pathogens in ticks, which are blood-feeding on migratory birds in northern Europe, and the current study adds new information to this subject. Our study is the first one to report the presence of *Babesia* species in ticks collected from birds in Sweden.

The prevalence of *Babesia* spp. detected in this study (2.4%) is on a similar level as what has been reported in ticks collected from birds in other countries in northern Europe, i.e., Norway, northern Germany, and north-western Russia (1.0–4.7%) [33–35]. In those studies, the *Babesia* species were identified as *B. venatorum*, *B. microti*, and *B. divergens*. In our study, *B. venatorum* was the most prevalent species and was detected in nymphs as well as in larvae of *I. ricinus*. Both *B. venatorum* [8] and *B. divergens* [36] are known to be transovarially transmitted. Thus, their presence in tick larvae should not be taken as an indication that the pathogen was derived from feeding upon the avian hosts. To elucidate that, one would need to investigate the presence of *Babesia* spp. in blood taken directly from the birds. Our data suggest that *B. capreoli* is also transovarially transmitted in *I. ricinus* ticks, since it was present in a larval tick. This was expected, given the close phylogenetic relationship between *B. divergens* and *B. capreoli* and since it is a trait regarded as common to all members of the genus *Babesia *sensu stricto [19].

*B. microti*, on the other hand, is not considered to be vertically transmitted [19], and was only found in nymphs. In contrast to our results, Franke et al. [37] and Hildebrandt et al. [34] recorded *B. microti* in *I. ricinus* larvae removed from several passerine bird species in Germany. We are not aware of any other reports where tick larvae removed from birds have been shown to harbour *B. microti.* On the contrary, the different taxa included in the *B. microti* complex are generally considered to have different species of small mammals, specifically rodents and shrews, as their main vertebrate reservoirs [13, 19, 38–43]. It might be possible that the *B. microti* infections recorded by Franke et al. [37] and Hildebrandt et al. [34] in larvae of *I. ricinus* were acquired by co-feeding transmission from nymphal or adult ticks that had previously fed on *B. microti*-infected mammals. However, experimental evidence from North America supports the notion that birds can act as reservoirs of *B. microti*: Hersh and colleagues tested 10 North American mammal species and four bird species for reservoir competency, i.e., capacity to infect the main vector in North America, *Ixodes scapularis*, with *B. microti*. They found reservoir competence levels of > 17% in white-footed mouse (*Peromyscus leucopus*), racoon (*Procyon lotor*), short-tailed shrew (*Blarina brevicauda*), and eastern chipmunk (*Tamias striatus*), and < 6% but > 0% in all other species, including all four bird species tested including three species belonging to the Turdidae family [44]. There is significant genetic heterogeneity among strains of *B. microti* [41–43, 45]. Therefore, further genetic investigations are necessary as well as studies to explore whether bird species even in Europe are systemically infected, competent reservoirs (transmission hosts) for any of these taxa of “*B. microti*”.

The tick species *H. punctata* and *Hy. marginatum* have been reported to harbour different *Babesia* species, where *B. motasi*, *B. bovis*, *B. bigemina*, *B. caballi* and *B. major* have been detected in *H. punctata*, and *B. microti* has been detected in *Hy. marginatum* [46–49]. A serological survey of sheep indicated that *B. motasi* may be present on the island of Gotland, south-eastern Sweden, where it is presumably vectored by *H. punctata* [12]. To our knowledge, neither *H. punctata* nor *Hy. marginatum* ticks removed from birds have been investigated previously for the presence of *Babesia* species. However, in the present study, only *I. ricinus* ticks were positive for *Babesia*. This is not surprising, given the overall low prevalence of *Babesia* spp. detected in the *I. ricinus* ticks, and the small number of specimens of the other tick species analysed.

In this study, the *Babesia*-positive ticks were found on eight common passerine bird species. Given the location, all these individuals are considered to be on active migration. Four of the species—European robin (*Erithacus rubecula*), common starling (*Sturnus vulgaris*), common blackbird (*Turdus merula*), and Eurasian wren (*Troglodytes troglodytes*)—are common and widespread short-distance migrant (or partial migrant) species that occur in a range of habitats, including in the vicinity of humans, in gardens, forests, and pastures. The other four species are obligatory long-distance migrants wintering in sub-Saharan Africa, predominantly West Africa, and include tree pipit (*Anthus trivialis*), common redstart (*Phoenicurus phoenicurus*), common whitethroat (*Sylvia communis*), and lesser whitethroat (*Sylvia curruca*), and although common, they are less associated with human settlements [50]. Interestingly, 17 of the bird individuals infested by *Babesia*-positive ticks were captured during their northward spring migration, suggesting a potential role of these birds as disseminators of *Babesia* protozoan parasites into Sweden. Although the prevalence of *Babesia* spp. was low, the sheer number of birds involved in migration would imply a significant number of introductions of infected ticks on an annual basis. Quantifying this number would require larger sample sizes and a wider sampling of host species. However, the presence of infected ticks in common passerine species that occur in habitats also frequented by humans and domesticated animals suggests that the risk of encountering potentially *Babesia*-infective ticks exists. However, the risk to humans resulting from these introductions remains speculative, especially in relation to the enzootic occurrence of these pathogens.

Two of the three *Babesia* species, i.e., *B. venatorum* and *B. microti*, detected in this study are previously known to cause human disease. In Europe, the most common cause of clinical human babesiosis is *B. divergens*, which typically is diagnosed in immunocompromised individuals and often gives rise to a severe illness [16, 51]. Human disease caused by *B. divergens* has also been reported in immunocompetent patients [52]. A few cases of *B. microti* and *B. venatorum* infection have also been reported in Europe [21, 22, 24, 53]. In contrast to *B. divergens*, *B. venatorum*, and *B. microti, B. capreoli* is considered not to be human-pathogenic [32].

In southern Sweden, a prevalence of 2.5% for *B. microti* and/or *B. divergens* antibodies among healthy individuals was recorded, and an even higher seroprevalence (16.3%) among seropositive *Borrelia burgdorferi* sensu lato patients was detected [54]. Despite the high seroprevalence, only two cases of human babesiosis have so far been reported in Sweden [18, 53]. Thus, the risk of developing severe babesiosis after an *I. ricinus* tick bite among healthy individuals in Sweden appears to be low [29]. However, as pointed out previously by others [19, 25, 54], we cannot exclude that this potentially severe infection is underdiagnosed.

## Conclusions

This is the first study showing the presence of *B. venatorum*, *B. microti*, and *B. capreoli* in ticks removed from birds in Sweden. The study also reveals that zoonotic *Babesia* species are present in ticks infesting migratory birds in south-eastern Sweden, which could lead to dissemination of these tick-borne microorganisms into new areas.

## Supplementary Information

**Additional file 1:** The aligned *Babesia* nucleotide sequences based on PCR-products.

## Data Availability

The data supporting the conclusions of this article are included within the article. Raw data can be shared with researchers upon a specific request.
