# Supplementary material for: Three Babesia species in Ixodes ricinus ticks from migratory birds in Sweden
Source: Parasit Vectors. 2021 Apr 1;14:183. doi: 10.1186/s13071-021-04684-8 (PMC8017608; doi:10.1186/s13071-021-04684-8)
Supplement: Supplementary file 1 — Additional file 1: The aligned Babesia nucleotide sequences based on PCR-products. [file 13071_2021_4684_MOESM1_ESM.docx]

**Additional file 1. Aligned *Babesia 18S* ribosomal RNA gene (partial sequence) nucleotide sequences based on PCR-products**

The identifier line, which begins with '>', gives the name of the **sample ID_microorganism_gene target_tick species_developmental stage of tick_bird species that the tick was removed from**

**>84884_Babesia venatorum_18S ribosomal RNA gene, partial sequence_Ixodes ricinus_nymph_Turdus merula**

ATGATGGTGACCTAAACCCTCACCAGAGTAACAATTGGAGGGCAAGTCTGGTGCCAGCAGCCGCGGTAATTCCAGCTCCAATAGCGTATATTAAACTTGTTGCAGTTAAAAAGCTCGTAGTTGAATTTCTGCGTTATCGAGTTATTGACTCTTGTCTTTAATCGATTTCGCTTTTGGGATTTATCCCTTTTTACTTTGAGAAAATTAGAGTGTTTCAAGCAGACTTTTGTCTTGAATACTTCAGCATGGAATAATAGAGTAGGACTTTGGTTCTATTTTGTTGGTTTTTGAACCTTAGTAATGGTTAATAGGAACGGTTGGGGGCATTCGTATTTAACTGTCAGAGGTGAAATTCTTAGATTTGTTAAAGACGAACTACTGCGAAAGCATTTGCCAAGGACGTTTCCATTAATCAAGAACGAAAGTTAGGGGATCGAAGACGATCAGATACCGTCGTAGTCCTAACCATAAACTA

**>84913_Babesia microti_18S ribosomal RNA gene, partial sequence_Ixodes ricinus_nymph_Turdus merula**

GGAATGATGGGAATCTAAACCCTTCCCAGAGTATCAATTGGAGGGCAAGTCTGGTGCCAGCAGCCGCGGTAATTCCAGCTCCAATAGCGTATATTAAAGTTGTTGCAGTTAAGAAGCTCGTAGTTGAATTTCTGCCTTGTCATTAATCTCGCTTCCGAGCGTTTTTTTATTGGCTTGGCATCTTCTGGATTTGGTGCCTTCGGGTACTATTTTCCAGGATTTACTTTGAGAAAACTAGAGTGTTTCAAACAGGCATTCGCCTTGAATACTACAGCATGGAATAATGAAGTAGGACTTTGGTTCTATTTTGTTGGTTATTGAGCCAGAGTAATGGTTAATAGGAGCAGTTGGGGGCATTCGTATTTAACTGTCAGAGGTGAAATTCTTAGATTTGTTAAAGACGAACTACTGCGAAAGCATTTGCCAAGGATGTTTTCATTAATCAAGAACGAAAGTTAGGGGATCGAAGACGATCAGATACCGTCGTAGTCC

**>85008_Babesia venatorum_18S ribosomal RNA gene, partial sequence_Ixodes ricinus_nymph_Erithacus rubecula**

GTCTTGTAATTGGAATGATGGTGACCTAAACCCTCACCAGAGTAACAATTGGAGGGCAAGTCTGGTGCCAGCAGCCGCGGTAATTCCAGCTCCAATAGCGTATATTAAACTTGTTGCAGTTAAAAAGCTCGTAGTTGAATTTCTGCGTTATCGAGTTATTGACTCTTGTCTTTAATCGATTTCGCTTTTGGGATTTATCCCTTTTTACTTTGAGAAAATTAGAGTGTTTCAAGCAGACTTTTGTCTTGAATACTTCAGCATGGAATAATAGAGTAGGACTTTGGTTCTATTTTGTTGGTTTTTGAACCTTAGTAATGGTTAATAGGAACGGTTGGGGGCATTCGTATTTAACTGTCAGAGGTGAAATTCTTAGATTTGTTAAAGACGAACTACTGCGAAAGCATTTGCCAAGGACGTTTCCATTAATCAAGAACGAAAGTTAGGGGATCGAAGACGATCAGATACCGTCGTAGTCCTAACCATAAACTA

**>85585_Babesia microti_18S ribosomal RNA gene, partial sequence_Ixodes ricinus_nymph_Sylvia curruca**

GTCTTGTAATTGGAATGATGGGAATCTAAACCCTTCCCAGAGTATCAATTGGAGGGCAAGTCTGGTGCCAGCAGCCGCGGTAATTCCAGCTCCAATAGCGTATATTAAAGTTGTTGCAGTTAAGAAGCTCGTAGTTGAATTTCTGCCTTGTCATTAATCTCGCTTCCGAGCGTTTTTTTATTGGCTTGGCATCTTCTGGATTTGGTGCCTTCGGGTACTATTTTCCAGGATTTACTTTGAGAAAACTAGAGTGTTTCAAACAGGCATTCGCCTTGAATACTACAGCATGGAATAATGAAGTAGGACTTTGGTTCTATTTTGTTGGTTATTGAGCCAGAGTAATGGTTAATAGGAGCAGTTGGGGGCATTCGTATTTAACTGTCAGAGGTGAAATTCTTAGATTTGTTAAAGACGAACTACTGCGAAAGCATTTGCCAAGGATGTTTTCATTAATCAAGAACGAAAGTTAGGGGATCGAAGACGATCAGATACCGTCGTAGTCCTAACCATAAACTA

**>85608_Babesia microti_18S ribosomal RNA gene, partial sequence_Ixodes ricinus_nymph_ Phoenicurus phoenicurus**

GTCTTGTAATTGGAATGATGGGAATCTAAACCCTTCCCAGAGTATCAATTGGAGGGCAAGTCTGGTGCCAGCAGCCGCGGTAATTCCAGCTCCAATAGCGTATATTAAAGTTGTTGCAGTTAAGAAGCTCGTAGTTGAATTTCTGCCTTGTCATTAATCTCGCTTCCGAGCGTTTTTTTATTGGCTTGGCATCTTCTGGATTTGGTGCCTTCGGGTACTATTTTCCAGGATTTACTTTGAGAAAACTAGAGTGTTTCAAACAGGCATTCGCCTTGAATACTACAGCATGGAATAATGAAGTAGGACTTTGGTTCTATTTTGTTGGTTATTGAGCCAGAGTAATGGTTAATAGGAGCAGTTGGGGGCATTCGTATTTAACTGTCAGAGGTGAAATTCTTAGATTTGTTAAAGACGAACTACTGCGAAAGCATTTGCCAAGGATGTTTTCATTAATCAAGAACGAAAGTTAGGGGATCGAAGACGATCAGATACCGTCGTAGTCCTA

**>85695_Babesia microti_18S ribosomal RNA gene, partial sequence_Ixodes ricinus_nymph_ Phoenicurus phoenicurus**

GTCTTGTAATTGGAATGATGGGAATCTAAACCCTTCCCAGAGTATCAATTGGAGGGCAAGTCTGGTGCCAGCAGCCGCGGTAATTCCAGCTCCAATAGCGTATATTAAAGTTGTTGCAGTTAAGAAGCTCGTAGTTGAATTTCTGCCTTGTCATTAATCTCGCTTCCGAGCGTTTTTTTATTGGCTTGGCATCTTCTGGATTTGGTGCCTTCGGGTACTATTTTCCAGGATTTACTTTGAGAAAACTAGAGTGTTTCAAACAGGCATTCGCCTTGAATACTACAGCATGGAATAATGAAGTAGGACTTTGGTTCTATTTTGTTGGTTATTGAGCCAGAGTAATGGTTAATAGGAGCAGTTGGGGGCATTCGTATTTAACTGTCAGAGGTGAAATTCTTAGATTTGTTAAAGACGAACTACTGCGAAAGCATTTGCCAAGGATGTTTTCATTAATCAAGAACGAAAGTTAGGGGATCGAAGACGATCAGATACCGTCGTAGTCCTA

**>85720_Babesia venatorum_18S ribosomal RNA gene, partial sequence_Ixodes ricinus_larva_Erithacus rubecula**

GTCTTGTAATTGGAATGATGGTGACCTAAACCCTCACCAGAGTAACAATTGGAGGGCAAGTCTGGTGCCAGCAGCCGCGGTAATTCCAGCTCCAATAGCGTATATTAAACTTGTTGCAGTTAAAAAGCTCGTAGTTGAATTTCTGCGTTATCGAGTTATTGACTCTTGTCTTTAATCGATTTCGCTTTTGGGATTTATCCCTTTTTACTTTGAGAAAATTAGAGTGTTTCAAGCAGACTTTTGTCTTGAATACTTCAGCATGGAATAATAGAGTAGGACTTTGGTTCTATTTTGTTGGTTTTTGAACCTTAGTAATGGTTAATAGGAACGGTTGGGGGCATTCGTATTTAACTGTCAGAGGTGAAATTCTTAGATTTGTTAAAGACGAACTACTGCGAAAGCATTTGCCAAGGACGTTTCCATTAATCAAGAACGAAAGTTAGGGGATCGAAGACGATCAGATACCGTCGTAGTCCTA

**>85919_Babesia venatorum_18S ribosomal RNA gene, partial sequence_Ixodes ricinus_larva_Erithacus rubecula**

GTCTTGTAATTGGAATGATGGTGACCTAAACCCTCACCAGAGTAACAATTGGAGGGCAAGTCTGGTGCCAGCAGCCGCGGTAATTCCAGCTCCAATAGCGTATATTAAACTTGTTGCAGTTAAAAAGCTCGTAGTTGAATTTCTGCGTTATCGAGTTATTGACTCTTGTCTTTAATCGATTTCGCTTTTGGGATTTATCCCTTTTTACTTTGAGAAAATTAGAGTGTTTCAAGCAGACTTTTGTCTTGAATACTTCAGCATGGAATAATAGAGTAGGACTTTGGTTCTATTTTGTTGGTTTTTGAACCTTAGTAATGGTTAATAGGAACGGTTGGGGGCATTCGTATTTAACTGTCAGAGGTGAAATTCTTAGATTTGTTAAAGACGAACTACTGCGAAAGCATTTGCCAAGGACGTTTCCATTAATCAAGAACGAAAGTTAGGGGATCGAAGACGATCAGATACCGTCGTAGTCCTA

**>85947_Babesia venatorum_18S ribosomal RNA gene, partial sequence_Ixodes ricinus_larva_Troglodytes troglodytes**

GTCTTGTTAAAAGGAATGATGGTGACCTAAACCCTCACCAGAGTAACAATTGGAGGGCAAGTCTGGTGCCAGCAGCCGCGGTAATTCCAGCTCCAATAGCGTATATTAAACTTGTTGCAGTTAAAAAGCTCGTAGTTGAATTTCTGCGTTATCGAGTTATTGACTCTTGTCTTTAATCGATTTCGCTTTTGGGATTTATCCCTTTTTACTTTGAGAAAATTAGAGTGTTTCAAGCAGACTTTTGTCTTGAATACTTCAGCATGGAATAATAGAGTAGGACTTTGGTTCTATTTTGTTGGTTTTTGAACCTTAGTAATGGTTAATAGGAACGGTTGGGGGCATTCGTATTTAACTGTCAGAGGTGAAATTCTTAGATTTGTTAAAGACGAACTACTGCGAAAGCATTTGCCAAGGACGTTTCCATTAATCAAGAACGAAAGT TAGGGGATCGAAGACGATCAGATACCGTCGTAGTCCTA

**>85132_Babesia venatorum_18S ribosomal RNA gene, partial sequence_Ixodes ricinus_larva_Erithacus rubecula**

GTCTTGTAATTGGAATGATGGTGACCTAAACCCTCACCAGAGTAACAATTGGAGGGCAAGTCTGGTGCCAGCAGCCGCGGTAATTCCAGCTCCAATAGCGTATATTAAACTTGTTGCAGTTAAAAAGCTCGTAGTTGAATTTCTGCGTTATCGAGTTATTGACTCTTGTCTTTAATCGATTTCGCTTTTGGGATTTATCCCTTTTTACTTTGAGAAAATTAGAGTGTTTCAAGCAGACTTTTGTCTTGAATACTTCAGCATGGAATAATAGAGTAGGACTTTGGTTCTATTTTGTTGGTTTTTGAACCTTAGTAATGGTTAATAGGAACGGTTGGGGGCATTCGTATTTAACTGTCAGAGGTGAAATTCTTAGATTTGTTAAAGACGAACTACTGCGAAAGCATTTGCCAAGGACGTTTCCATTAATCAAGAACGAAAGTTAGGGGATCGAAGACGATCAGATACCGTCGTAGTCCTA

**>85197_Babesia microti_18S ribosomal RNA gene, partial sequence_Ixodes ricinus_nymph_Erithacus rubecula**

GTCTTGTAATTGGAATGATGGGAATCTAAACCCTTCCCAGAGTATCAATTGGAGGGCAAGTCTGGTGCCAGCAGCCGCGGTAATTCCAGCTCCAATAGCGTATATTAAAGTTGTTGCAGTTAAGAAGCTCGTAGTTGAATTTCTGCCTTGTCATTAATCTCGCTTCCGAGCGTTTTTTTATTGGCTTGGCATCTTCTGGATTTGGTGCCTTCGGGTACTATTTTCCAGGATTTACTTTGAGAAAACTAGAGTGTTTCAAACAGGCATTCGCCTTGAATACTACAGCATGGAATAATGAAGTAGGACTTTGGTTCTATTTTGTTGGTTATTGAGCCAGAGTAATGGTTAATAGGAGCAGTTGGGGGCATTCGTATTTAACTGTCAGAGGTGAAATTCTTAGATTTGTTAAAGACGAACTACTGCGAAAGCATTTGCCAAGGATGTTTTCATTAATCAAGAACGAAAGTTAGGGGATCGAAGACGATCAGATACCGTCGTAGTCCTA

**>85232_Babesia venatorum_18S ribosomal RNA gene, partial sequence_Ixodes ricinus_nymph_Erithacus rubecula**

GTCTTGTAATTGGAATGATGGTGACCTAAACCCTCACCAGAGTAACAATTGGAGGGCAAGTCTGGTGCCAGCAGCCGCGGTAATTCCAGCTCCAATAGCGTATATTAAACTTGTTGCAGTTAAAAAGCTCGTAGTTGAATTTCTGCGTTATCGAGTTATTGACTCTTGTCTTTAATCGATTTCGCTTTTGGGATTTATCCCTTTTTACTTTGAGAAAATTAGAGTGTTTCAAGCAGACTTTTGTCTTGAATACTTCAGCATGGAATAATAGAGTAGGACTTTGGTTCTATTTTGTTGGTTTTTGAACCTTAGTAATGGTTAATAGGAACGGTTGGGGGCATTCGTATTTAACTGTCAGAGGTGAAATTCTTAGATTTGTTAAAGACGAACTACTGCGAAAGCATTTGCCAAGGACGTTTCCATTAATCAAGAACGAAAGTTAGGGGATCGAAGACGATCAGATACCGTCGTAGTCCTA

**>85236_Babesia venatorum_18S ribosomal RNA gene, partial sequence_Ixodes ricinus_larva_Troglodytes troglodytes**

GTCTTGTAATTGGAATGATGGTGACCTAAACCCTCACCAGAGTAACAATTGGAGGGCAAGTCTGGTGCCAGCAGCCGCGGTAATTCCAGCTCCAATAGCGTATATTAAACTTGTTGCAGTTAAAAAGCTCGTAGTTGAATTTCTGCGTTATCGAGTTATTGACTCTTGTCTTTAATCGATTTCGCTTTTGGGATTTATCCCTTTTTACTTTGAGAAAATTAGAGTGTTTCAAGCAGACTTTTGTCTTGAATACTTCAGCATGGAATAATAGAGTAGGACTTTGGTTCTATTTTGTTGGTTTTTGAACCTTAGTAATGGTTAATAGGAACGGTTGGGGGCATTCGTATTTAACTGTCAGAGGTGAAATTCTTAGATTTGTTAAAGACGAACTACTGCGAAAGCATTTGCCAAGGACGTTTCCATTAATCAAGAACGAAAGTTAGGGGATCGAAGACGATCAGATACCGTCGTAGTCCTAACCA

**>85252_Babesia microti_18S ribosomal RNA gene, partial sequence_Ixodes ricinus_nymph_Anthus trivialis**

GTCTTGTAATTGGAATGATGGGAATCTAAACCCTTCCCAGAGTATCAATTGGAGGGCAAGTCTGGTGCCAGCAGCCGCGGTAATTCCAGCTCCAATAGCGTATATTAAAGTTGTTGCAGTTAAGAAGCTCGTAGTTGAATTTCTGCCTTGTCATTAATCTCGCTTCCGAGCGTTTTTTTATTGGCTTGGCATCTTCTGGATTTGGTGCCTTCGGGTACTATTTTCCAGGATTTACTTTGAGAAAACTAGAGTGTTTCAAACAGGCATTCGCCTTGAATACTACAGCATGGAATAATGAAGTAGGACTTTGGTTCTATTTTGTTGGTTATTGAGCCAGAGTAATGGTTAATAGGAGCAGTTGGGGGCATTCGTATTTAACTGTCAGAGGTGAAATTCTTAGATTTGTTAAAGACGAACTACTGCGAAAGCATTTGCCAAGGATGTTTTCATTAATCAAGAACGAAAGTTAGGGGATCGAAGACGATCAGATACCGTCGTAGTCCTA

**>85275_Babesia microti_18S ribosomal RNA gene, partial sequence_Ixodes ricinus_nymph_ Anthus trivialis**

GTCTTGTAATTGGAATGATGGGAATCTAAACCCTTCCCAGAGTATCAATTGGAGGGCAAGTCTGGTGCCAGCAGCCGCGGTAATTCCAGCTCCAATAGCGTATATTAAAGTTGTTGCAGTTAAGAAGCTCGTAGTTGAATTTCTGCCTTGTCATTAATCTCGCTTCCGAGCGTTTTTTTATTGGCTTGGCATCTTCTGGATTTGGTGCCTTCGGGTACTATTTTCCAGGATTTACTTTGAGAAAACTAGAGTGTTTCAAACAGGCATTCGCCTTGAATACTACAGCATGGAATAATGAAGTAGGACTTTGGTTCTATTTTGTTGGTTATTGAGCCAGAGTAATGGTTAATAGGAGCAGTTGGGGGCATTCGTATTTAACTGTCAGAGGTGAAATTCTTAGATTTGTTAAAGACGAACTACTGCGAAAGCATTTGCCAAGGATGTTTTCATTAATCAAGAACGAAAGTTAGGGGATCGAAGACGATCAGATACCGTCGTAGTCCTA

**>85317_Babesia venatorum_18S ribosomal RNA gene, partial sequence_Ixodes ricinus_larva_Erithacus rubecula**

GTCTTGTAATTGGAATGATGGTGACCTAAACCCTCACCAGAGTAACAATTGGAGGGCAAGTCTGGTGCCAGCAGCCGCGGTAATTCCAGCTCCAATAGCGTATATTAAACTTGTTGCAGTTAAAAAGCTCGTAGTTGAATTTCTGCGTTATCGAGTTATTGACTCTTGTCTTTAATCGATTTCGCTTTTGGGATTTATCCCTTTTTACTTTGAGAAAATTAGAGTGTTTCAAGCAGACTTTTGTCTTGAATACTTCAGCATGGAATAATAGAGTAGGACTTTGGTTCTATTTTGTTGGTTTTTGAACCTTAGTAATGGTTAATAGGAACGGTTGGGGGCATTCGTATTTAACTGTCAGAGGTGAAATTCTTAGATTTGTTAAAGACGAACTACTGCGAAAGCATTTGCCAAGGACGTTTCCATTAATCAAGAACGAAAGTTAGGGGATCGAAGACGATCAGATACCGTCGTAGTCCTA

**>85361_Babesia venatorum_18S ribosomal RNA gene, partial sequence_Ixodes ricinus_larva_Phoenicurus phoenicurus**

GTCTTGTAATTGGAATGATGGTGACCTAAACCCTCACCAGAGTAACAATTGGAGGGCAAGTCTGGTGCCAGCAGCCGCGGTAATTCCAGCTCCAATAGCGTATATTAAACTTGTTGCAGTTAAAAAGCTCGTAGTTGAATTTCTGCGTTATCGAGTTATTGACTCTTGTCTTTAATCGATTTCGCTTTTGGGATTTATCCCTTTTTACTTTGAGAAAATTAGAGTGTTTCAAGCAGACTTTTGTCTTGAATACTTCAGCATGGAATAATAGAGTAGGACTTTGGTTCTATTTTGTTGGTTTTTGAACCTTAGTAATGGTTAATAGGAACGGTTGGGGGCATTCGTATTTAACTGTCAGAGGTGAAATTCTTAGATTTGTTAAAGACGAACTACTGCGAAAGCATTTGCCAAGGACGTTTCCATTAATCAAGAACGAAAGTTAGGGGATCGAAGACGATCAGATACCGTCGTAGTCCTA

**>85362_Babesia venatorum_18S ribosomal RNA gene, partial sequence_Ixodes ricinus_nymph_ Sylvia communis**

GTCTTGTAATTGGAATGATGGTGACCTAAACCCTCACCAGAGTAACAATTGGAGGGCAAGTCTGGTGCCAGCAGCCGCGGTAATTCCAGCTCCAATAGCGTATATTAAACTTGTTGCAGTTAAAAAGCTCGTAGTTGAATTTCTGCGTTATCGAGTTATTGACTCTTGTCTTTAATCGATTTCGCTTTTGGGATTTATCCCTTTTTACTTTGAGAAAATTAGAGTGTTTCAAGCAGACTTTTGTCTTGAATACTTCAGCATGGAATAATAGAGTAGGACTTTGGTTCTATTTTGTTGGTTTTTGAACCTTAGTAATGGTTAATAGGAACGGTTGGGGGCATTCGTATTTAACTGTCAGAGGTGAAATTCTTAGATTTGTTAAAGACGAACTACTGCGAAAGCATTTGCCAAGGACGTTTCCATTAATCAAGAACGAAAGTTAGGGGATCGAAGACGATCAGATACCGTCGTAGTCCTA

**>85368_Babesia venatorum_18S ribosomal RNA gene, partial sequence_Ixodes ricinus_larva_Phoenicurus phoenicurus**

GTCTTGTAATTGGAATGATGGTGACCTAAACCCTCACCAGAGTAACAATTGGAGGGCAAGTCTGGTGCCAGCAGCCGCGGTAATTCCAGCTCCAATAGCGTATATTAAACTTGTTGCAGTTAAAAAGCTCGTAGTTGAATTTCTGCGTTATCGAGTTATTGACTCTTGTCTTTAATCGATTTCGCTTTTGGGATTTATCCCTTTTTACTTTGAGAAAATTAGAGTGTTTCAAGCAGACTTTTGTCTTGAATACTTCAGCATGGAATAATAGAGTAGGACTTTGGTTCTATTTTGTTGGTTTTTGAACCTTAGTAATGGTTAATAGGAACGGTTGGGGGCATTCGTATTTAACTGTCAGAGGTGAAATTCTTAGATTTGTTAAAGACGAACTACTGCGAAAGCATTTGCCAAGGACGTTTCCATTAATCAAGAACGAAAGTTAGGGGATCGAAGACGATCAGATACCGTCGTAGTC

**>85382_Babesia capreoli_18S ribosomal RNA gene, partial sequence_Ixodes ricinus_larva_Phoenicurus phoenicurus**

TGGAATGATGGTGACCTAAACCCTCACCAGAGTAACAATTGGAGGGCAAGTCTGGTGCCAGCAGCCGCGGTAATTCCAGCTCCAATAGCGTATATTAAACTTGTTGCAGTTAAAAAGCTCGTAGTTGAATTTTTGCGTGGTGTTAATATTGACTGATGTCGAGATTGCACTTCGCTTTTGGGATTTTTCCCTTTTTACTTTGAGAAAATTAGAGTGTTTCAAGCAGACTTTTGTCTTGAATACTTCAGCATGGAATAATAGAGTAGGACTTTGGTTCTATTTTGTTGGTTTGTGAACCTTAGTAATGGTTAATAGGAACGGTTGGGGGCATTCGTATTTAACTGTCAGAGGTGAAATTCTTAGATTTGTTAAAGACGAACTACTGCGAAAGCATTTGCCAAGGACGTTTTCATTAATCAAGAACGAAAGTTAGGGGATCGAAGACGATCAGATACCGTCGTAGTCCTAACCATAAACTA

**>85433_Babesia microti_18S ribosomal RNA gene, partial sequence_Ixodes ricinus_nymph_ Phoenicurus phoenicurus**

GTCTTGTAATTGGAATGATGGGAATCTAAACCCTTCCCAGAGTATCAATTGGAGGGCAAGTCTGGTGCCAGCAGCCGCGGTAATTCCAGCTCCAATAGCGTATATTAAAGTTGTTGCAGTTAAGAAGCTCGTAGTTGAATTTCTGCCTTGTCATTAATCTCGCTTCCGAGCGTTTTTTTATTGGCTTGGCATCTTCTGGATTTGGTGCCTTCGGGTACTATTTTCCAGGATTTACTTTGAGAAAACTAGAGTGTTTCAAACAGGCATTCGCCTTGAATACTACAGCATGGAATAATGAAGTAGGACTTTGGTTCTATTTTGTTGGTTATTGAGCCAGAGTAATGGTTAATAGGAGCAGTTGGGGGCATTCGTATTTAACTGTCAGAGGTGAAATTCTTAGATTTGTTAAAGACGAACTACTGCGAAAGCATTTGCCAAGGATGTTTTCATTAATCAAGAACGAAAGTTAGGGGATCGAAGACGATCAGATACCGTCGTAGTCCTA

**>85380_Babesia microti_18S ribosomal RNA gene, partial sequence_Ixodes ricinus_nymph_ Phoenicurus phoenicurus**

GTCTTGTAATTGGAATGATGGGAATCTAAACCCTTCCCAGAGTATCAATTGGAGGGCAAGTCTGGTGCCAGCAGCCGCGGTAATTCCAGCTCCAATAGCGTATATTAAAGTTGTTGCAGTTAAGAAGCTCGTAGTTGAATTTCTGCCTTGTCATTAATCTCGCTTCCGAGCGTTTTTTTATTGGCTTGGCATCTTCTGGATTTGGTGCCTTCGGGTACTATTTTCCAGGATTTACTTTGAGAAAACTAGAGTGTTTCAAACAGGCATTCGCCTTGAATACTACAGCATGGAATAATGAAGTAGGACTTTGGTTCTATTTTGTTGGTTATTGAGCCAGAGTAATGGTTAATAGGAGCAGTTGGGGGCATTCGTATTTAACTGTCAGAGGTGAAATTCTTAGATTTGTTAAAGACGAACTACTGCGAAAGCATTTGCCAAGGATGTTTTCATTAATCAAGAACGAAAGTTAGGGGATCGAAGACGATCAGATACCGTCGTAGTCCTA

**>85436_Babesia venatorum_18S ribosomal RNA gene, partial sequence_Ixodes ricinus_nymph_Sturnus vulgaris**

GTCTTGTAATTGGAATGATGGTGACCTAAACCCTCACCAGAGTAACAATTGGAGGGCAAGTCTGGTGCCAGCAGCCGCGGTAATTCCAGCTCCAATAGCGTATATTAAACTTGTTGCAGTTAAAAAGCTCGTAGTTGAATTTCTGCGTTATCGAGTTATTGACTCTTGTCTTTAATCGATTTCGCTTTTGGGATTTATCCCTTTTTACTTTGAGAAAATTAGAGTGTTTCAAGCAGACTTTTGTCTTGAATACTTCAGCATGGAATAATAGAGTAGGACTTTGGTTCTATTTTGTTGGTTTTTGAACCTTAGTAATGGTTAATAGGAACGGTTGGGGGCATTCGTATTTAACTGTCAGAGGTGAAATTCTTAGATTTGTTAAAGACGAACTACTGCGAAAGCATTTGCCAAGGACGTTTCCATTAATCAAGAACGAAAGTTAGGGGATCGAAGACGATCAGATACCGTCGTAGTCCTA

**>85450_Babesia venatorum_18S ribosomal RNA gene, partial sequence_Ixodes ricinus_larva_Turdus merula**

GTCTTGTAATTGGAATGATGGTGACCTAAACCCTCACCAGAGTAACAATTGGAGGGCAAGTCTGGTGCCAGCAGCCGCGGTAATTCCAGCTCCAATAGCGTATATTAAACTTGTTGCAGTTAAAAAGCTCGTAGTTGAATTTCTGCGTTATCGAGTTATTGACTCTTGTCTTTAATCGATTTCGCTTTTGGGATTTATCCCTTTTTACTTTGAGAAAATTAGAGTGTTTCAAGCAGACTTTTGTCTTGAATACTTCAGCCATGGAATAATAGAGTAGGACCCTTTGGTTCTTATTTTGTTGGTTTTTGAACCTTAGTAATGGTTAATAGGAACGGTTGGGGGCATTCGTATTTAACTGTCAGAGGTGAAATTCTTAGATTTGTTAAAGACGAACTACTGCGAAAGCATTTGCCAAGGACGTTTCCATTAATCAAGAACGAAAGTTAGGGGATCGAAGACGATCAGATACCGTCGTAGTCCTAACCATAAACTA

**>85451_Babesia venatorum_18S ribosomal RNA gene, partial sequence_Ixodes ricinus_nymph_Turdus merula**

GTCTTGTAATTGGAATGATGGTGACCTAAACCCTCACCAGAGTAACAATTGGAGGGCAAGTCTGGTGCCAGCAGCCGCGGTAATTCCAGCTCCAATAGCGTATATTAAACTTGTTGCAGTTAAAAAGCTCGTAGTTGAATTTCTGCGTTATCGAGTTATTGACTCTTGTCTTTAATCGATTTCGCTTTTGGGATTTATCCCTTTTTACTTTGAGAAAATTAGAGTGTTTCAAGCAGACTTTTGTCTTGAATACTTCAGCATGGAATAATAGAGTAGGACTTTGGTTCTATTTTGTTGGTTTTTGAACCTTAGTAATGGTTAATAGGAACGGTTGGGGGCATTCGTATTTAACTGTCAGAGGTGAAATTCTTAGATTTGTTAAAGACGAACTACTGCGAAAGCATTTGCCAAGGACGTTTCCATTAATCAAGAACGAAAGTTAGGGGATCGAAGACGATCAGATACCGTCGTAGTCCTAACCATAAACTA

**>86038_Babesia microti_18S ribosomal RNA gene, partial sequence_Ixodes ricinus_nymph_Turdus merula**

GTCTTGTAATTGGAATGATGGTGACCTAAACCCTTCCCAGAGTATCAATTGGAGGGCAAGTCTGGTGCCAGCAGCCGCGGTAATTCCAGCTCCAATAGCGTATATTAAAGTTGTTGCAGTTAAGAAGCTCGTAGTTGAATTTCTGCCTTGTCATTAATCTCGCTTCCGAGCGTTTTTTTATTGGCTTGGCATCTTCTGGATTTGGTGCCTTCGGGTACTATTTTCCAGGATTTACTTTGAGAAAACTAGAGTGTTTCAAACAGGCATTCGCCTTGAATACTACAGCATGGAATAATGAAGTAGGACTTTGGTTCTATTTTGTTGGTTATTGAGCCAGAGTAATGGTTAATAGGAGCAGTTGGGGGCATTCGTATTTAACTGTCAGAGGTGAAATTCTTAGATTTGTTAAAGACGAACTACTGCGAAAGCATTTGC
